# Supplementary material for: Glycoprotein and Lipoprotein Profiles Assessed by 1H-NMR and Its Relation to Ascending Aortic Dilatation in Bicuspid Aortic Valve Disease
Source: J Clin Med. 2022 Dec 31;12(1):332. doi: 10.3390/jcm12010332 (PMC9821550; doi:10.3390/jcm12010332)
Supplement: Supplementary file 1 [file jcm-12-00332-s001.zip › jcm-2094310-supplementary.pdf]

Journal of
Clinical Medicine

MDPI

Table S1. Correlations between ascending aorta diameter and glycoprotein and lipoprotein profiles

	**AAo Diameter**	
	**Pearson's Correlation**	**p value**
**GlycB (µmol/L)**	**0.197***	**0.015**
**GlycF (µmol/L)**	0.099	0.223
**GlycA (µmol/L)**	0.133	0.102
**H/W GlyB**	**0.176***	**0.030**
**H/W GlycA**	0.097	0.237
**HDL-C (mg/dL)**	-0.043	0.596
**HDL-P (µmol/L)**	0.020	0.810
**HDL-TG (mg/dL)**	0.124	0.127
**HDL-TG/HDL-C**	0.144	0.076
**HDL-Z (nm)**	-0.079	0.335
**IDL-C (mg/dL)**	**0.320****	**<0.001**
**IDL-TG (mg/dL)**	**0.309****	**<0.001**
**IDL-TG/IDL-C**	**-0.289****	**<0.001**
**Large HDL-P (µmol/L)**	0.165*	0.043
**Large LDL-P (nmol/L)**	0.168*	0.038
**Large VLDL-P (nmol/L)**	0.115	0.158
**LDL-C (mg/dL)**	**0.247****	**0.002**
**LDL-P (nmol/L)**	**0.285****	**<0.001**
**LDL-TG (mg/dL)**	**0.226****	**0.005**
**LDL-TG/LDL-C**	0.016	0.848
**LDL-Z (nm)**	-0.152	0.062
**Medium HDL-P (µmol/L)**	-0.046	0.570
**Medium LDL-P (nmol/L)**	**0.197***	**0.015**
**Medium VLDL-P (nmol/L)**	0.085	0.296
**remCholesterol (mg/dL)**	**0.226****	**0.005**
**Small HDL-P (µmol/L)**	0.048	0.557
**Small LDL-P (nmol/L)**	**0.320****	**<0.001**
**Small VLDL-P (nmol/L)**	**0.185***	**0.022**
**TOTAL-C (mg/dL)**	**0.267****	**0.001**
**TOTAL-TGs (mg/dL)**	**0.175***	**0.031**
**VLDL-C (mg/dL)**	0.151	0.063
**VLDL-P (nmol/L)**	**0.169***	**0.037**
**VLDL-TG (mg/dL)**	0.147	0.070
**VLDL-TG/VLDL-C**	0.026	0.748
**VLDL-Z (nm)**	**-0.196***	**0.015**

AAo: Ascending Aorta; remCholesterol: Remnant cholesterol; TG: Triglycerides; C: Cholesterol; HDL: High Density Lipoprotein IDL: Intermediale Density Lipoprotein; LDL: Low Density

Lipoprotein; VLDL: Very Low Density Lipoprotein; P: Particles; H/W: Height/Weight. * Significant values ($p < 0.05$), ** Significant values ($p < 0.01$).
